# Voltage-gated sodium channels as targets for pyrethroid insecticides

**DOI:** 10.1007/s00249-016-1195-1

**Published:** 2017-01-09

**Authors:** Linda M. Field, T. G. Emyr Davies, Andrias O. O’Reilly, Martin S. Williamson, B. A. Wallace

**Affiliations:** 1 Rothamsted Research, Herts, Harpenden, AL5 2JQ UK; 2 Birkbeck College, University of London, London, WC1E 7HX UK; 3 Liverpool John Moores University, Liverpool, L3 3AF UK

**Keywords:** Insecticides, Mode of action, Modelling, Pyrethroids, Resistance

## Abstract

The pyrethroid insecticides are a very successful group of compounds that have been used extensively for the control of arthropod pests of agricultural crops and vectors of animal and human disease. Unfortunately, this has led to the development of resistance to the compounds in many species. The mode of action of pyrethroids is known to be via interactions with the voltage-gated sodium channel. Understanding how binding to the channel is affected by amino acid substitutions that give rise to resistance has helped to elucidate the mode of action of the compounds and the molecular basis of their selectivity for insects vs mammals and between insects and other arthropods. Modelling of the channel/pyrethroid interactions, coupled with the ability to express mutant channels in oocytes and study function, has led to knowledge of both how the channels function and potentially how to design novel insecticides with greater species selectivity.

## Introduction

There is much concern among the public and policymakers that pesticides are damaging non-target organisms and polluting the environment. Whilst in some cases these views may be justified, they must be balanced against the need to produce sufficient high-quality, nutritious and safe food. Given that the world population is currently about 7 billion, and that this is predicted to increase to 11 billion by 2100, more food will have to be produced, with little opportunity to expand the growing areas. Up to 40% of global crop yields are lost to pests and diseases every year, and these losses could double without pesticides (ECPA 2016), so we clearly need to be able to control these threats in order to achieve sustainable food production. This must be done alongside the conservation of biodiversity, which is particularly challenging with regard to insects, where we often need to control crop pests without damaging other insects such as pollinators and providers of other ecosystem services. Control of pests largely relies on chemical insecticides, and although there are possibilities for alternative control measures, these are unlikely to replace chemistry in the foreseeable future.

When man first started to cultivate crops, the only options for pest control were manual removal of pests or physical protection from infestation, and this remained so until insecticidal compounds such as arsenic and copper were used, followed by the first availability of synthetic compounds in the 1940s. These were the organochlorines (notably DDT), later replaced by organophosphates (OPs) and carbamates in the 1950s, which lasted until the 1970s. These compounds played an important role in insect control but were also toxic to non-target organisms, even mammals, which gave rise to understandable worries about their use. An important breakthrough in crop protection came in the mid-1970s with the new synthetic pyrethroids, offering good insect control with very low toxicity to mammals (for reviews see Casida 2010; Soderlund 2014).

The first commercial pyrethroids (discovered at Rothamsted), and based on the known insecticidal properties of pyrethrins from the pyrethrum daisy, were bioresmethrin, permethrin, cypermethrin and deltamethrin. These all had much higher activity towards flies than did pyrethrin I, with deltamethrin, for example, being 1400 times more active. They also had much lower toxicity to mammals, with deltamethrin being approximately 100-fold less toxic than pyrethrin I. By 2002, deltamethrin had the highest global sales of any pyrethroid, at $208 million per year, and pyrethroids became the most widely used insecticidal compounds. This remained the case until the 1990s, when they started to become less effective as resistance developed and a new group of chemicals, the neonicotinoids, came onto the market. However, pyrethroids are still widely used in both agriculture and vector control.

### Mode of action of pyrethroids

**Fig. 1:**
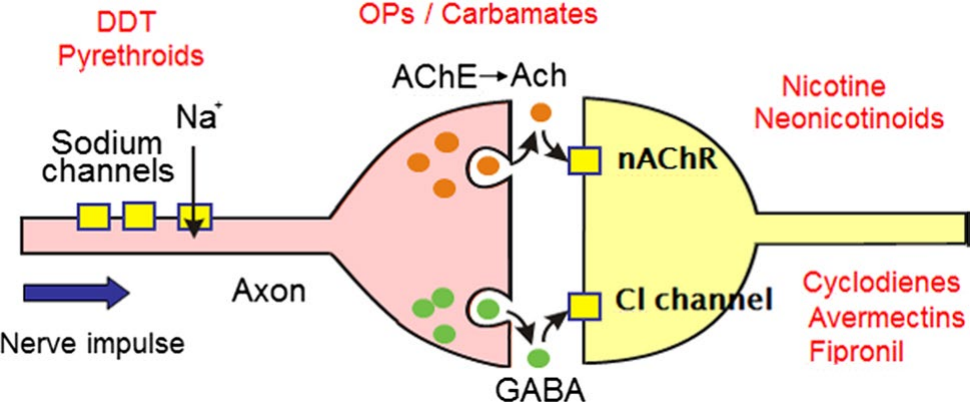
Diagrammatic representation of two neurons and an intervening synapse, showing the sites of action of the most commonly used classes of insecticide

Most synthetic insecticides target nervous system proteins, as summarised in Fig. 1. Pyrethroids, like DDT, bind to the voltage-gated sodium channel (VGSC), preventing its transition from an activated (ion-conducting) to an inactivated (non-conducting) state (Davies et al. 2007). As a result, the membranes of electrically excitable cells become persistently depolarised and the insect is paralysed and dies quickly, often exhibiting a ‘knock-down’ response. Thus the first signs of the development of resistance to these compounds became known as ‘knock-down resistance’ (kdr). Many of the mutations responsible for resistance have now been identified in a number of insect species, including *kdr* [encoding an amino acid substitution at position 1014 (*Musca domestica* VGSC numbering) in the VGSC] and the more potent form of resistance, *super*-*kdr* (encoding an additional substitution at amino acid position 918) (Davies et al. 2007). Substitution at position 929 also affects interactions of both DDT and pyrethroids with the channel. These mutations have a profound effect on the control of many important crop pest species (aphids, beetles, moths, weevils) and vectors of human disease (mosquitoes), and an understanding of the genetic changes involved has played an important role in developing diagnostics for monitoring resistance in the field.

**Fig. 2:**
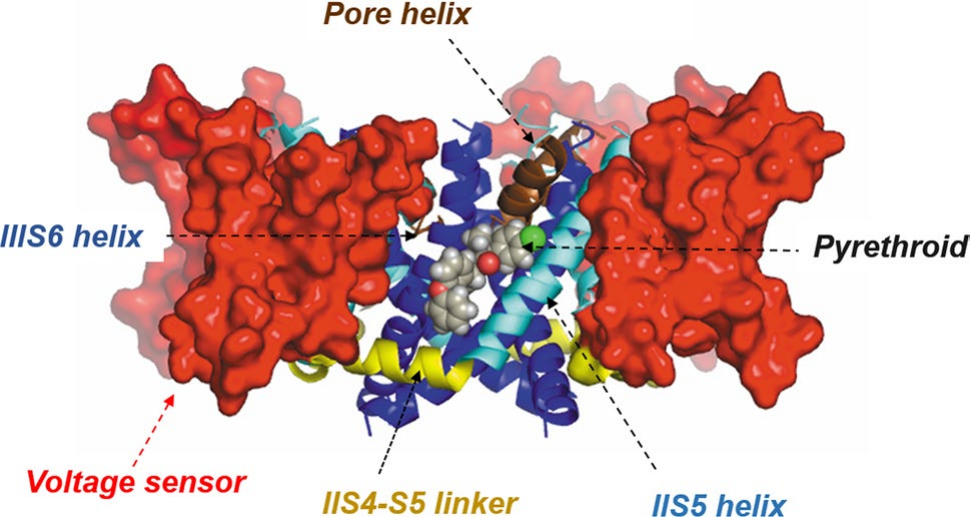
Homology model for housefly sodium channel (adapted from O’Reilly et al. 2006)

An important step in understanding how pyrethroids interact with the VGSC came when a homology model for the housefly channel was used to predict the binding site for the compounds (O’Reilly et al. 2006). This model was developed based on the Shaker rat-brain K_v_1.2 structure (Long et al. 2005), which was the first voltage-gated ion channel crystallised adopting a native conformation. The K_v_1.2 structure revealed how each voltage-sensor domain (comprising S1–S4 transmembrane helices) was connected to the ion-conducting pore module (S5–S6 helices) by a helical S4–S5 linker. K_v_1.2 shares over 20% sequence identity with the domain II transmembrane region of the housefly VGSC and when modelled, the S4–S5 linker and the S5 and S6 helices of domain II, together with the S6 helix of domain III, were found to shape a hydrophobic pocket that faced the lipid bilayer. This pocket was predicted to be accessible to lipid-soluble insecticidal compounds, and the structures of DDT and different pyrethroids were computationally docked to study their binding interactions (Fig. 2). Understanding how pyrethroids interact with the VGSC in turn allowed us to predict how mutations that change the channel would affect the efficacy of different pyrethroids, and has helped us understand why mammals are much less sensitive to pyrethroids and why some pyrethroids have differing toxicity towards insects and ticks/mites.

### Effect of resistance mutations on insecticide binding to the voltage-gated sodium channel

**Fig. 3:**
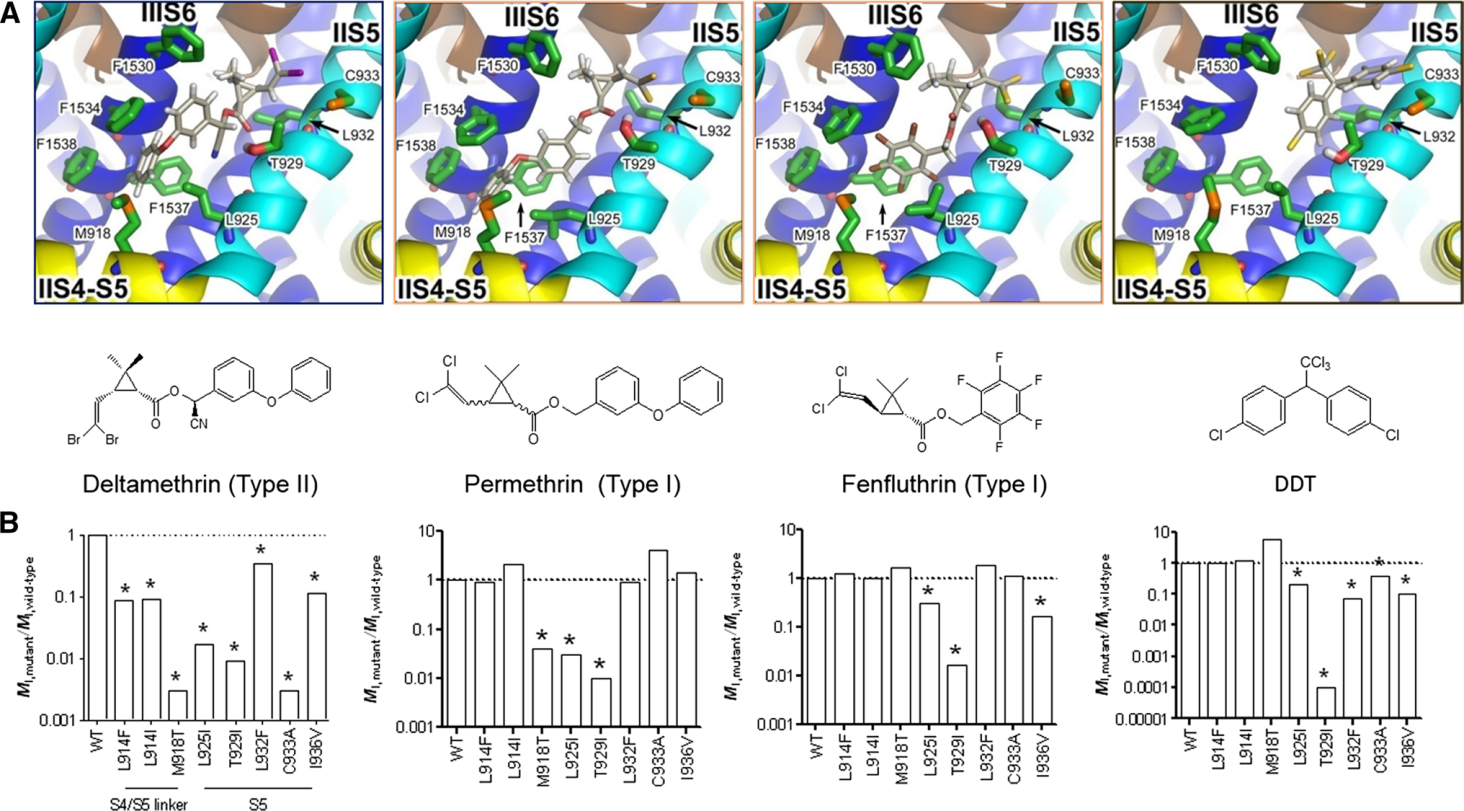
Predicted binding of three pyrethroids and DDT to the binding site of the VGSC adapted from Usherwood et al. (2007) and the effect of amino acid substitutions on the binding of three pyrethroids and DDT to VGSCs expressed in oocytes. Adapted from O’Reilly et al. (2006)

The VGSC model (O’Reilly et al. 2006) predicts that changes in some amino acids will affect the binding of some pyrethroids and not others. For example, a change at T929, which is in a region of the binding site where deltamethrin, permethrin, fenfluthrin and DDT bind, should confer resistance to all four compounds, whereas a change at M918, which is at the bottom of the pocket and away from where fenfluthrin (a relatively short chain pyrethroid) and DDT are predicted to bind, should confer resistance to permethrin and deltamethrin only (Fig. 3). These predictions have been tested experimentally by expressing VGCSs with a range of amino acid substitutions in oocytes and using electrophysiological recordings to monitor changes in channel function in the presence of deltamethrin, permethrin, fenfluthrin and DDT. In Fig. 3 the effect of each mutation relative to the wild-type channel (WT) is shown, with a value lower than the WT indicating less binding i.e. a resistant channel.

These results show that, as predicted by the model, T929I confers resistance to all four compounds, whereas M918T provides resistance to deltamethrin and permethrin but not fenfluthrin or DDT. Such information can be used to inform the best use of different compounds in the field and avoid costly use of chemistry unlikely to work. Earlier electrophysiological experiments on isolated segmental nerves and neuromuscular junctions of *M. domestica* larvae with kdr (L1014F) and kdr/super-kdr (L1014F/M918T), had also intimated a mitigation of resistance to fenfluthrin when M918T was present (Pepper and Osborne 1993). The relative effectiveness of fenfluthrin (and related short-chain multi-halogenated benzyl pyrethroids such as tefluthrin and transfluthrin) against L1014F/M918T *M. domestica* vs L1014F flies was also demonstrated very recently in bioassays using live insects (Sun et al. 2016), further supporting the predictions of the O’Reilly model.

An alternative, dual-receptor site model for binding of pyrethroids and DDT was also recently proposed (Du et al. 2015, 2016; Zhorov and Dong 2017), whereby simultaneous binding of two molecules to receptor sites PyR1 (O’Reilly et al. 2006) and PyR2 are needed to lock the sodium channel in the open state. The PyR2 and PyR1 sites are proposed to be located in domain interfaces I/II and II/III, respectively, and are arranged quasi-symmetrically. At each site the pyrethroids bind between four helices: L45, S5 and two S6 helices from adjacent domains (Du et al. 2015). One difference between these models is that L1014F is firmly localized within the PyR2 site of the Du model, whereas in the original O’Reilly model L1014F is postulated to affect pyrethroid binding via an indirect (allosteric) impact; the effect of L1014F is to slow VGSC opening, which is predicted to consequently reduce the rate of PyR1 formation, thus limiting pyrethroid binding availability and conferring kdr. A second difference is that the orientation of bound pyrethroids within each pocket is reversed (throwing into question why M918T would be ineffective against compounds such as fenfluthrin), and the pyrethroids penetrate significantly deeper into the protein domain in PyR2. The localisation of DDT within the binding pockets is similarly translocated from the top to the bottom of the pocket. Curiously, if such a dual-receptor site for pyrethroids exists in the VGSC, very few natural resistance mutations, apart from those clustered around the kdr site on the IIS6 helix, have been identified that localise within PyR2, in contrast to the profusion identified for PyR1 (Rinkevich et al. 2013). There is clearly a need to address the conflicts presented by the two models in terms of how pyrethroids and DDT interact with the channel to exert their effects and the exact role of mutations in mitigating the effects of these compounds on the channel.

### Why are mammals much less susceptible to pyrethroids?

**Fig. 4:**
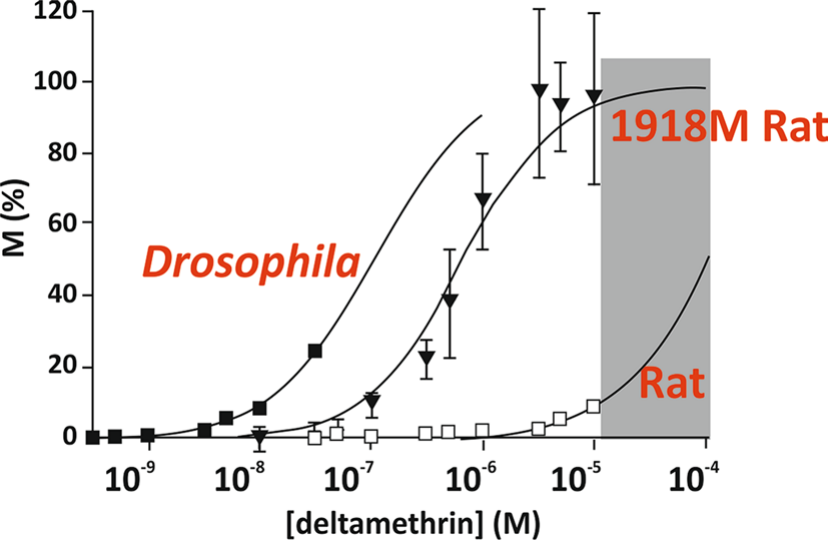
Response of VGSCs from *Drosophila melanogaster*, rat and a mutated rat channel to deltamethrin at a range of concentrations (adapted from Vais et al. 2000)

Ever since pyrethroids were developed, there have been questions as to why they are toxic to insects but much less so to mammals. There are a number of potential reasons, including the fact that lipid solubility may favour entry through the insect cuticle, differences in detoxification and a negative temperature coefficient (they are more effective below 25 °C) (Soderlund 2012; Narahashi et al. 2007). However, there is good evidence that there is a direct effect of differences in sodium channel sequences which make pyrethroids less able to bind to mammalian channels. The knowledge that the amino acid at position 918 in the channels makes a difference to sensitivity, and that gene sequences reveal that arthropods generally have a methionine (M) at this position but mammals and other organisms such as fish do not, led to the hypothesis that the methionine at 918 is essential for toxicity (Soderlund 2012; Vais et al. 2000). This was tested by taking the gene encoding the rat VGSC, mutating it so that the resulting channel had a methionine at 918 [instead of the usual isoleucine (I)] and testing the binding of deltamethrin on channels expressed in oocytes. Figure 4 shows the results for an insect channel (*Drosophila melanogaster*) with M at 918, the native rat channel with I at 918 and the mutant rat channel with the single I918 M substitution. This clearly shows that changing just this one amino acid makes the rat VGSC much more sensitive to deltamethrin. This selectivity demonstrates that it is possible to have very selective compounds, and that if we can fully understand how insecticides bind to their targets, then there is the possibility of ‘designer’ compounds that ideally target one insect over another. This principle has already been demonstrated for drug design (for a review see Fernandez-Ballester et al. 2011). Although no one has yet designed an insecticide to kill one insect and not another, there are big differences in toxicity already in compounds belonging to the same class of insecticides; thus it requires only 0.0015 µg deltamethrin to kill a bee but 10 µg of another pyrethroid, *Tau*-fluvalinate. The same compounds also show very different effects on insects and ticks/mites (see next section).

### Why do some pyrethroids have differing toxicity towards insects and acarines?

It has been observed that the pyrethroid *Tau*-fluvalinate is much more toxic to ticks and mites (acarines) than it is to insects, and this has had important practical implications for control of *Varroa destructor* mites in bee hives where *Tau*-fluvalinate has been widely used. One difference between the VGSC of acarines and insects is at amino acid position 933, which is a cysteine (C) in insects and a glycine (G), valine (V) or alanine (A) in acarines. Modelling of the interactions of these VGSCs with the pyrethroids suggests that the C in insect channels obstructs binding of *Tau*-fluvalinate, making it a relatively poor insecticide. However, when the C is replaced by comparatively smaller amino acids as in acarines, *Tau*-fluvalinate then has the necessary room to fit and so binds more tightly, making it a good acaricide (O’Reilly et al. 2012). Again, this provides a better understanding of how VGSCs work and how specificity can be achieved.

### Aphid VGSCs

Our recently published work (Amey et al. 2015) using aphid genome resources to identify VGSC sequences has identified a number of unusual properties of the aphid channel that are not present in the channels of other insects. The aphid VGSC is a unique heterodimeric channel, with an atypical ion selectivity filter and, unusual for insect channels, is highly insensitive to tetrodotoxin. This channel most likely arose by adaptation (fission) of an invertebrate ancestral mono- (hetero)-meric channel, possibly brought about by a chromosomal inversion event. It is the only identifiable VGSC homologue in aphid genomes, and the channel’s novel selectivity filter motif (DENS instead of the usual DEKA found in other eukaryotes) may result in a loss of sodium selectivity, as indicated experimentally in mutagenised *D. melanogaster* channels (Amey et al. 2015). These findings suggest that it may be possible to design compounds that would act on the aphid channel and not those of other insects.

Overall, the work discussed here on the VGSC as the target for insecticides has contributed to our wider understanding of how these channels have evolved and how they function. In the world of crop protection, where the aim is to have insecticides that kill pest and not non-target insects, these studies provide a basis for the potential design of more selective molecules.
